# Important regulatory function of transient receptor potential ankyrin 1 receptors in age-related learning and memory alterations of mice

**DOI:** 10.1007/s11357-019-00083-1

**Published:** 2019-07-20

**Authors:** Éva Borbély, Maja Payrits, Ágnes Hunyady, Gréta Mező, Erika Pintér

**Affiliations:** 1 Department of Pharmacology and Pharmacotherapy, Medical School, University of Pécs, Szigeti u.12., Pécs, 7624 Hungary; 2 Szentágothai Research Center, Center for Neuroscience, University of Pécs, Ifjúság u. 20, Pécs, 7624 Hungary

**Keywords:** TRPA1, Learning, Memory, Dementia

## Abstract

Expression of the transient receptor potential ankyrin 1 (TRPA1) receptor has been demonstrated not only in the dorsal root and trigeminal ganglia but also in different brain regions (e.g., hippocampus, hypothalamus, and cortex). However, data concerning their role in neurodegenerative and age-related diseases of the CNS is still indistinct. The aim of our study was to investigate the potential role of TRPA1 in a mouse model of senile dementia. For the investigation of changes during aging, we used male young (3–4-month-old) and old (18-month-old) wild-type (TRPA1^+/+^;WT) and TRPA1 receptor gene-deleted (TRPA1^−/−^) mice. Novel object recognition (NOR) test as well as Y maze (YM), radial arm maze (RAM), and Morris water maze (MWM) tests were used to assess the decline of memory and learning skills. In the behavioral studies, significant memory loss was detected in aged TRPA1^+/+^ mice with the NOR and RAM, but there was no difference measured by YM and MWM tests regarding the age and gene. TRPA1^−/−^ showed significantly reduced memory loss, which could be seen as higher discrimination index in the NOR and less exploration time in the RAM. Furthermore, young TRPA1^−/−^ animals showed significantly less reference memory error in the RAM and notably higher mobility in NOR, RAM, and YM compared with the age-matched WTs. Our present work has provided the first evidence that TRPA1 receptors mediate deteriorating effects in the old age memory decline. Understanding the underlying mechanisms could open new perspectives in the pharmacotherapy of dementia.

## Introduction

Transient receptor potential ankyrin 1 (TRPA1) is a polymodal, non-selective cation channel, which belongs to the TRP superfamily. It is widely expressed on neuronal and non-neuronal cell types (Nilius et al. 2012). The highest level of expression can be detected in the nervous system—primarily in the dorsal root and trigeminal ganglia as well as in capsaicin-sensitive sensory nerve endings (Zygmunt and Högestätt 2014). It plays a crucial role in several physiological and pathophysiological processes: pain sensation (Kádková et al. 2017; Hung and Tan 2018), inflammation (Koivisto et al. 2014; Choi and Di Nardo 2018), and cancer (Büch et al. 2018).

TRPA1 has also been shown in the central nervous system (CNS). It was detected in the hippocampus (Koch et al. 2011), nucleus supraopticus of the hypothalamus (Yokoyama et al. 2011), brain stem (Sun et al. 2009), and cortical neurons (Kheradpezhouh et al. 2017). Furthermore, TRPA1 receptors are expressed in astrocytes and play a role in the regulation of the intracellular calcium level resulting in the release of mediators (Shigetomi et al. 2011, 2013; Takizawa et al. 2018). Therefore, TRPA1 was recently intensively investigated in different central nervous system pathologies, e.g., multiple sclerosis (Sághy et al. 2016; Bölcskei et al. 2018) and Alzheimer’s disease (Lee et al. 2016). Additionally, due to its expression on endothelial cells, oxygen sensing, and calcium-regulating functions, TRPA1 has neuroprotective effects also in stroke (Pires and Earley 2018; Guerra et al. 2018). Moreover, TRPA1 seems to be a key contributor of ischemic myelin damage (Hamilton et al. 2016). These results strongly suggest that TRPA1 influences the neuroinflammatory/neurodegenerative alterations. However, results concerning the age-related expression and functional changes of TRPA1 are deficient. TRPA1 expression continuously increases in the mouse brain after birth but reaches a plateau in 2–3 weeks (Lee et al. 2017). Data about alterations of the receptor count/density in older animals are absolutely lacking. There is only one paper that compares the function of TRPA1 in young (3-month-old) and aged (24-month-old) mice. They have found that TRPA1 is a key mediator of nociceptor sensitization only in aged animals in an adjuvant-induced arthritis model (Garrison and Stucky 2014).

The mechanism of senile memory loss is still unclear. This type of dementia is usually moderate but it gradually leads to declined mental function, resulting in disability and loss of quality of life (Bowling et al. 2015). There are several neurodegenerative processes involved in senile dementia (Blalock et al. 2003). The most important factors are inflammatory responses (Hauss-Wegrzyniak et al. 2000; Andreasson et al. 2001; Franceschi and Campisi 2014), oxidative stress (Carney et al. 1991; Davies et al. 2017; Tan et al. 2018), mitochondrial dysfunction, and altered calcium homeostasis (Alzheimer’s Association Calcium Hypothesis Workgroup 2017; Müller et al. 2018; Sure et al. 2018). Investigation of the molecular background of this type of memory loss as well as the assessment of the effectiveness of different drug candidates is very complicated in rodents. Dementia occurs as a complex syndrome of cognitive, functional, and emotional alterations in humans (Ferrucci et al. 2018; Cansino et al. 2018); only some symptoms are reproducible in animal models (Mitchell et al. 2015; Wahl et al. 2017). Furthermore, the mortality, morbidity, and sensitivity to different interventions have much higher risk over 12 months in rodents (Snyder et al. 2016).

TRPA1 channel is a “promiscuous” receptor and can be activated by several electrophilic ligands (free radicals, inflammatory mediators, etc.) (Bandell et al. 2004; Nilius et al. 2012; Storozhuk and Zholos 2018; Pozsgai et al. 2019) which accumulate during aging. TRPA1 activation leads to increased intracellular calcium level with cellular damage aggravating dementia in a genetic mouse model of Alzheimer’s disease. Therefore, in the present study, we aimed to investigate the old age memory decline without any pharmacological or genetic manipulation. Another aim was to investigate the potential role of TRPA1 receptors in senile dementia in gene-deleted mice.

## Materials and methods

### Ethics

All experimental procedures were performed according to the 1998/XXVIII Act of the Hungarian Parliament on Animal Protection, Consideration Decree of Scientific Procedures of Animal Experiments (243/1988), Hungarian regulations (40/2013, II.14.), and Directive 2010/63/EU of the European Parliament. The studies were approved by the Ethics Committee on Animal Research of University of Pécs according to the Ethical Codex of Animal Experiments and license was given (license no. BA 02/2000-24/2016).

### Animals

Experiments were carried out using TRPA1 receptor gene-deficient mice (TRPA1^−/−^; KO) and their wild-type counterparts (TRPA1^+/+^; WT), respectively. TRPA1^−/−^ and TRPA^+/+^ mice were generated from an original pair of heterozygous mice and obtained from Prof. P. Geppetti (University of Florence, Italy). Offspring were genotyped and homozygous mice were selected for further breeding. The animals were bred and kept in the vivarium of the Department of Pharmacology and Pharmacotherapy of the University of Pécs at 24 °C and provided with standard rodent food and water ad libitum. The animals were maintained under conditions of 12-h light/dark cycle and were housed in groups of 3–5 in polycarbonate cages (530-cm^3^ floor space, 14-cm height) on wood shavings bedding. The animals had a 60-min acclimatization period prior to each experiment.

For the experiments, young (3–4-month-old) and old (18-month-old) male TRPA1^+/+^ and TRPA1^−/−^ mice were used. The number of animals was 6–12/group.

### Novel object recognition test (NOR)

For the assessment of the recognition memory, we used the well-known paradigm of the novel object recognition test (Morellini 2013). The examination lasted for 3 days. On the first, habituation day, animals could freely explore the 45 × 45 × 30 cm wooden box for a 5-min-long period, which can be considered as a simple open field test. In this regard, spontaneous locomotor activity can be detected and characterized by distance moved, velocity, time spent in the center, and entries to the central zone. On the second experimental day, mice were allowed to examine the two identical objects for 5 min. On the third day (24 h after the second trial), the animals could choose from one familiar and one novel object which has similar size but different shape and color. Mice explored the objects for 5 min. Behavior of animals was recorded and analyzed with Ethovision XT11 software (Noldus Information Technology, Netherlands). The obtained data were calculated and represented as follows: distance moved, time spent with moving, velocity (1st, 2nd, 3rd days); location preference = (time exploring the right identical object/total exploration time) × 100 (2nd day); recognition index = (time exploring the novel object/total exploration time) × 100 and discrimination index = difference in time exploring the novel and familiar objects/total exploration time, exploration time of the familiar and novel objects (3rd day).

### Radial arm maze test (RAM)

For the measurement of short- and long-term memory alterations, the radial arm maze test is a widely used, suitable method (Levin 1988). Three-day-long habituation and learning period was used before the test trial. During this time, mice have to learn where they can find the food pellets (Dustless Precision Pellets® 45 mg, Sucrose; BioServ, USA) placed into 4 previously chosen arms of the eight-arm radial maze (arms 5 × 35 cm, central platform diameter 5 cm). The trials lasted for 5 min or until the animals have found all the four food pellets, whichever came first. The learning ability was assessed on the 4th day of the experiment. Exploratory behavior was recorded and analyzed with Ethovision XT11 software (Noldus Information Technology, Netherlands). Data were calculated and represented as follows: working memory errors = entries into the baited arms that had already been visited during the same trial, referring to the short-term memory, and reference memory error = entries into empty arms, showing the status of long-term memory, velocity, and average exploration time = time spent in collecting all the pellets in the maze/total number of arm entries (Li et al. 2011; Zhang et al. 2000).

### Y maze test (YM)

For the assessment of spatial memory, Y maze test was used (D’Souza et al. 2015; Hughes 2004). Only one 5-min-long trial was performed in the equipment (three 35-cm-long × 5-cm-wide arms, stated as A, B, and C arms) and the spontaneous alternation (correct alternating behavior (ABC, ACB, BAC, BCA, CAB, CBA)/the number of arm entries minus two); distance moved, velocity, and total number of arm entries were determined on the basis of the video tracked by Ethovision XT11 software (Noldus Information Technology, Netherlands).

### Morris water maze test (MWM)

Morris water maze test is also widely used for both basic research and drug developmental purposes (D’Hooge and De Deyn 2001). It was originally developed for rats (Morris 1981), but later it was adapted to mice. In our hands, the following protocol was used: a circular pool was filled with water to hide the platform placed always at the same point of the pool. Visual clues were placed above the pool on the wall to help the orientation. Conditioning lasted for 3 days; each day, each animal performed 4 swimming sessions started from 4 different points of the pool (northeast (NE), southeast (SE), southwest (SW), and northwest (NW)). On the fourth day of the study, all mice had to perform the same 4 swimming sessions, and the time to find the platform (escape latency) was calculated as the mean of the 4 trials (Zhang et al. 2012). Swimming time was recorded and analyzed with Ethovision XT11 software (Noldus Information Technology, Netherlands).

### Heat maps

Different colors indicated by the heat maps show the average activity of the animal groups. It shows how and where the animals spent time during the tests. Cold colors (black, blue, and green) mean low activity. Warm colors (yellow, red) indicate high activity of the mice in different parts of the experimental area.

### Statistical analysis

Data in all experiments were expressed as mean ± SEM. Data are analyzed by two-way ANOVA followed by Fischer’s posttest. In case of location preference, one sample *t* test in comparison with 50% was used. In every case, *p* < 0.05 was considered significant. All statistical analyses were performed using Statistica software.

## Results

### Significantly attenuated memory loss was detected in old TRPA1^−/−^ animals by the NOR compared with the TRPA1^+/+^ respective controls

**Fig. 1:**
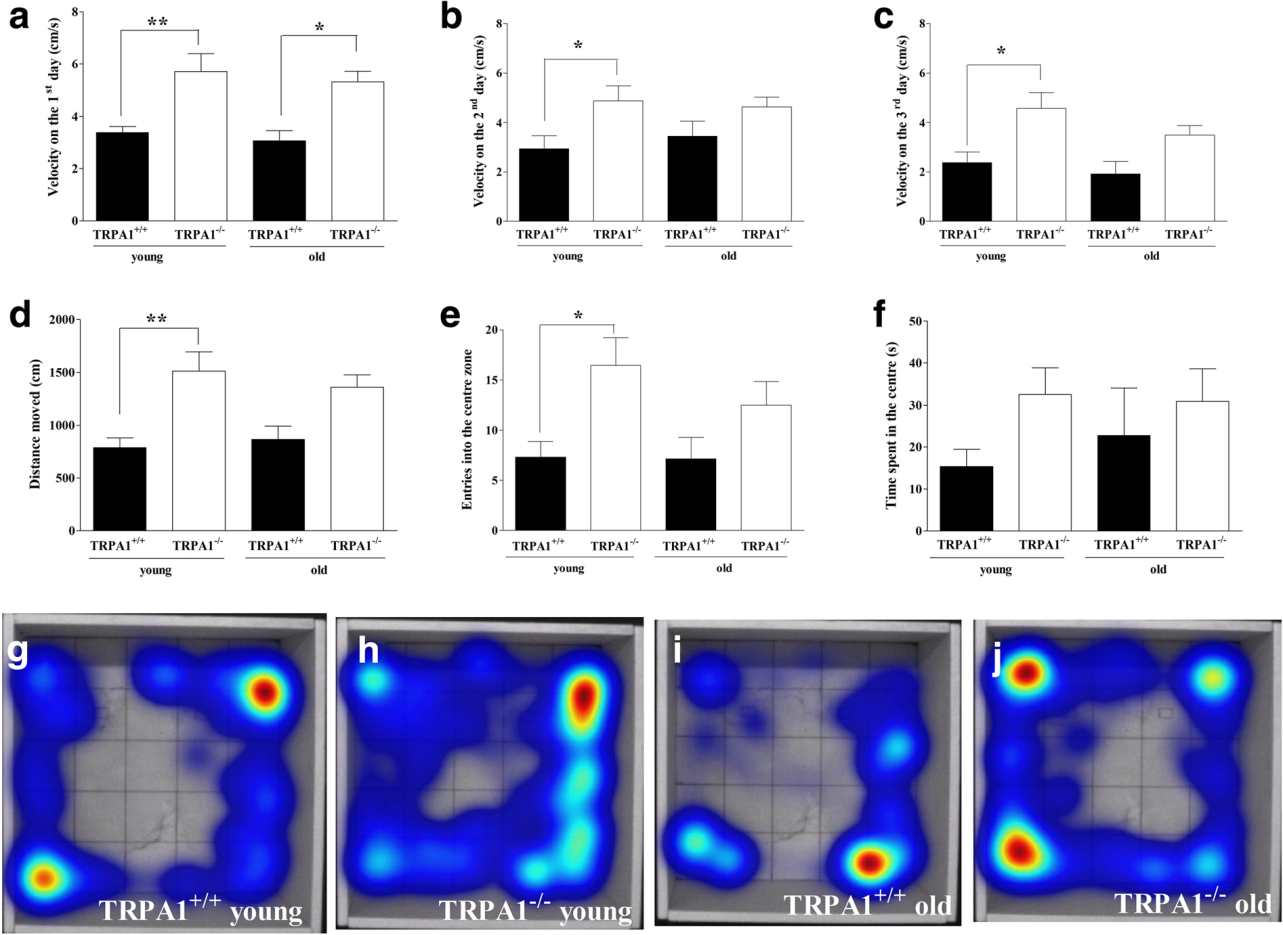
Age-dependent changes of the performance in the open field test (OFT)/1st day of novel object recognition (NOR) task in TRPA1^+/+^ and TRPA1^−/−^ young and old mice. Spontaneous locomotor activity was determined as velocity (**a**) and distance moved (**d**) as well as entries to the center zone (**e**). Anxiety level was assessed by the time spent in the center zone (**f**). Velocity was determined also on the 2nd (**b**) and 3rd (**c**) days of the NOR test. Data are presented as the mean ± SEM (*n* = 6–10) and were analyzed by two-way ANOVA followed by Fischer’s posttest (*p < 0.05, **p < 0.01). Demonstrative heat map pictures of the locomotor activity of TRPA1^+/+^ young (**g**) and old **(i**) as well as TRPA1^−/−^ young (**h**) and old (**j**) mice on the 1st day of the NOR test. Blue color represents the less visited parts of the experimental area; red color represents the most frequently visited parts of the experimental area

On the first day of the novel object recognition (NOR) test, when the animals can move around freely in the box, young TRPA1^−/−^ showed significantly higher mobility state. Young TRPA1^+/+^ mice moved 787.50 ± 92.37 cm with a velocity of 3.39 ± 0.23 cm/s, while these values in the case of the TRPA1^−/−^ animals were 1511.00 ± 184.80 cm and 5.71 ± 0.68 cm/s, respectively (Fig. 1a, d). Velocity of old gene-deleted animals was also higher (5.33 ± 0.39 cm/s) than that of the WT counterparts (3.07 ± 0.39 cm/s). Time spent in the center zone was not significantly different in the different groups and did not markedly alter with the age, but the entries in the center zone (TRPA1^+/+^ 7.33 ± 1.56, TRPA1^−/−^ 16.44 ± 2.80) showed the higher mobility state of the TRPA1^−/−^ animals again (Fig. 1e, f). Heat maps of the animals visualize the differences observed on the first day of the NOR. In case of both old and young TRPA1^−/−^ mice, the density maps show more colorful areas and the center zone is also covered by blue color which cannot be detected in the case of the respective WTs (Fig. 1g–j).

**Fig. 2:**
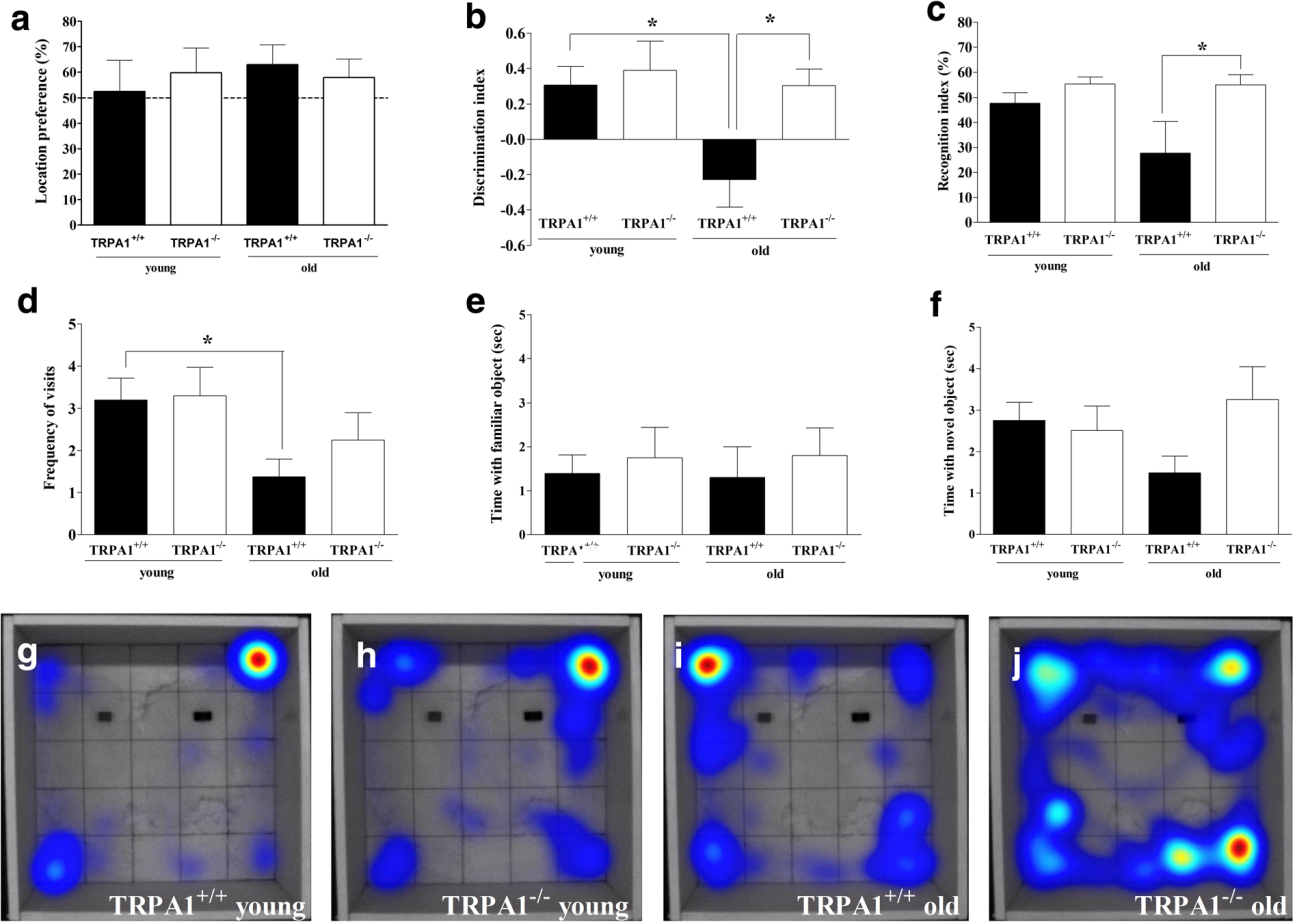
Age-dependent changes of the performance in the novel object recognition (NOR) task in TRPA1^+/+^ and TRPA1^−/−^ young and old mice. On the 2nd day of the test, time spent with exploring the two identical objects was demonstrated as location preference (**a**). Memory function was determined on the 3rd (test) day as discrimination index (**b**), recognition index (**c**), frequency of visiting the objects (**d**), and time spent with exploring the familiar (**e**) and novel (**f**) objects. Data are presented as the mean ± SEM (*n* = 6–10) and were analyzed by two-way ANOVA followed by Fischer’s posttest (*p < 0.05). In the case of location preference, one sample *t* test in comparison with 50% was used. Heat map pictures of the performance of TRPA1^+/+^ young (**g**) and old (**i**) as well as TRPA1^−/−^ young (**h**) and old (**j**) mice on the 3rd day of the NOR test. Blue color represents the less visited parts of the experimental area; red color represents the most frequently visited parts of the experimental area

On the second day, all animal groups showed no or minimal preference to one of the identical objects, so there was no significant location preference in any of the groups (Fig. 2a). The velocity was significantly higher in young TRPA1^−/−^ animals compared with the WT counterparts also on the 2nd and 3rd days of the experiment (Fig. 1b, c).

In old TRPA1^+/+^ mice, the frequency of visiting the novel object (1.37 ± 0.42) and the discrimination index (− 0.44 ± 0.25) was significantly deteriorated compared with the young TRPA1^+/+^ animals (3.20 ± 0.51 and 0.31 ± 0.10; Fig. 2b, d). In the gene-deleted animals, this memory loss cannot be detected; young and old TRPA1^−/−^ showed similar performance during the test trial (Fig. 2b–d). In comparison with old TRPA1^+/+^ and TRPA1^−/−^ mice, the NOR memory test has shown that both the discrimination (0.17 ± 0.05) and recognition indexes (55.08 ± 3.95) of the TRPA1^−/−^ group are significantly higher than those of the WTs (Fig. 2b, c). Heat maps (Fig. 2g-j) show the preference of the novel (right) object in every group except for the old TRPA1^+/+^. The most frequently visited place was very close to the novel object (top right corner) in the young groups.

### Young animals showed significantly better reference memory compared with the old animals, and attenuated memory loss was detected in old TRPA1 KO mice compared with the WT controls in the RAM

**Fig. 3:**
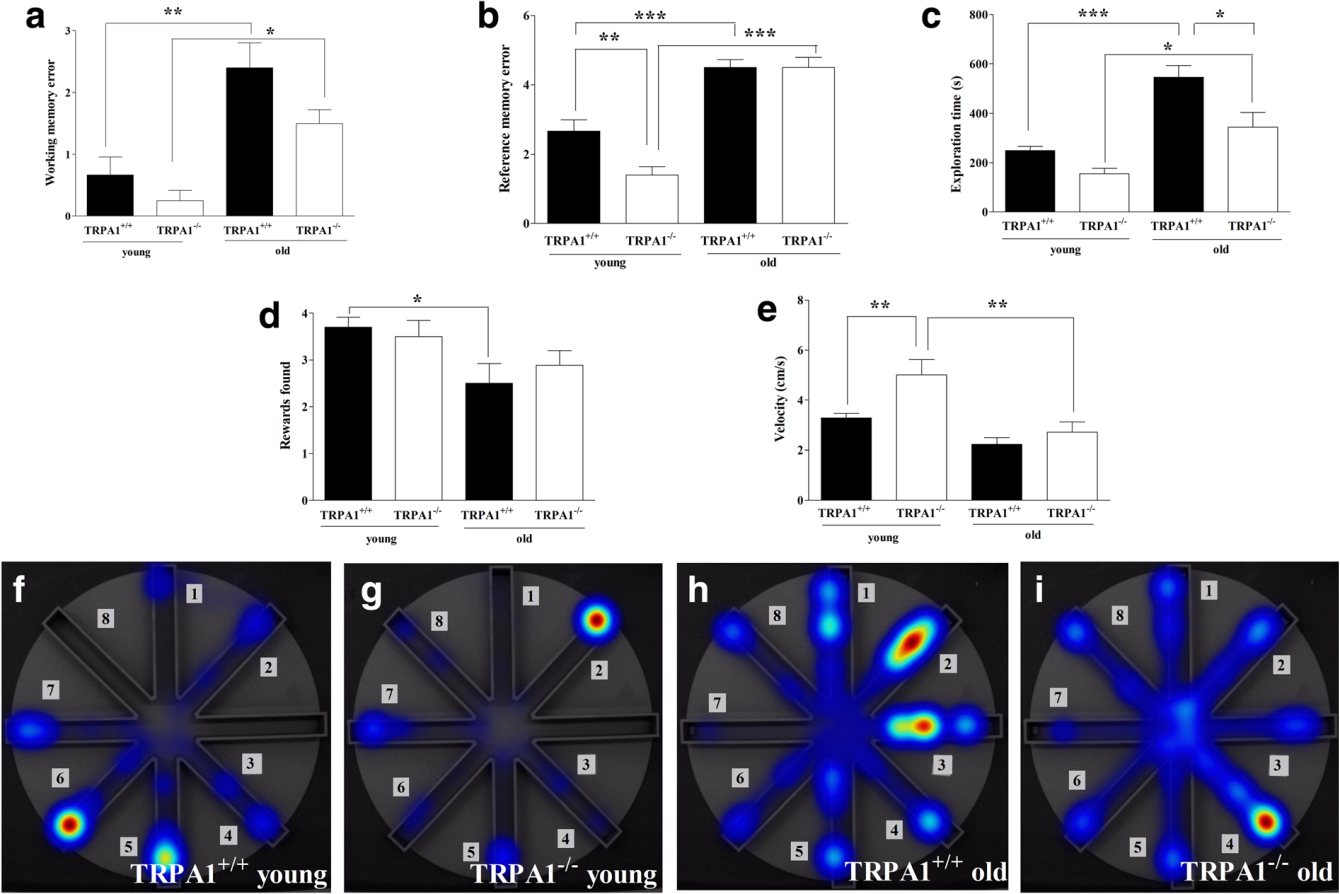
Age-dependent changes of the performance in the radial arm maze (RAM) task in TRPA1^+/+^ and TRPA1^−/−^ young and old mice. Memory function was determined as working memory error (**a**), reference memory error (**b**), average exploration time (**c**), and rewards found (**d**). Velocity of the animals (**e**) was also assessed during the measurement. Data are presented as the mean ± SEM (*n* = 6–10) and were analyzed by two-way ANOVA followed by Fischer’s posttest (*p < 0.05, **p < 0.01, ***p < 0.001). Heat map pictures of the performance of TRPA1^+/+^ young (**f**) and old (**h**) as well as TRPA1^−/−^ young (**g**) and old (**i**) mice on the 4th day of the RAM test. Blue color represents the less visited parts of the experimental area; red color represents the most frequently visited parts of the experimental area. Arms 1, 2, 5, and 7 were rewarded

The radial arm maze (RAM) test showed that old TRPA1^+/+^ and TRPA1^−/−^ mice need longer time for exploration than the young animals. Additionally, old TRPA1^−/−^ animals spend significantly less time (343.50 ± 60.17 s) with collecting the pellets than the old TRPA1^+/+^ (545.00 ± 47.45) mice (Fig. 3c). Furthermore, old TRPA1^+/+^ mice find significantly less rewards (2.50 ± 0.42) compared with the young WT counterparts (3.70 ± 0.21), which decline cannot be observed in the case of the gene-deleted animals (Fig. 3d). The velocity was significantly higher in the young TRPA1^−/−^ group compared with the WTs and remarkably decreased in aged TRPA1^−/−^ animals (Fig. 3e).

The reference memory error is significantly higher in the old groups (TRPA1^+/+^ 4.50 ± 0.22; TRPA1^−/−^ 4.50 ± 0.29) compared with their young counterparts (TRPA1^+/+^ 2.67 ± 0.33; TRPA1^−/−^ 1.40 ± 0.24). Furthermore, it was remarkably lower in young TRPA1^−/−^ mice than in WTs (Fig. 3b). The working memory error is significantly higher in old animals (TRPA1^+/+^ 2.40 ± 0.40; TRPA1^−/−^ 1.50 ± 0.22) compared with the young groups (TRPA1^+/+^ 0.67 ± 0.29; TRPA1^−/−^ 0.25 ± 0.16), and it is lower in both young and old TRPA1^−/−^ animals compared with their WTs, although it is not statistically significant (Fig. 3a). Heat maps clearly demonstrate that the young animals visited markedly less arm and made less errors compared with the old mice (Fig. 3f–i).

### Neither age nor TRPA1 gene deletion influenced the Y maze and Morris water maze test performance

**Fig. 4:**
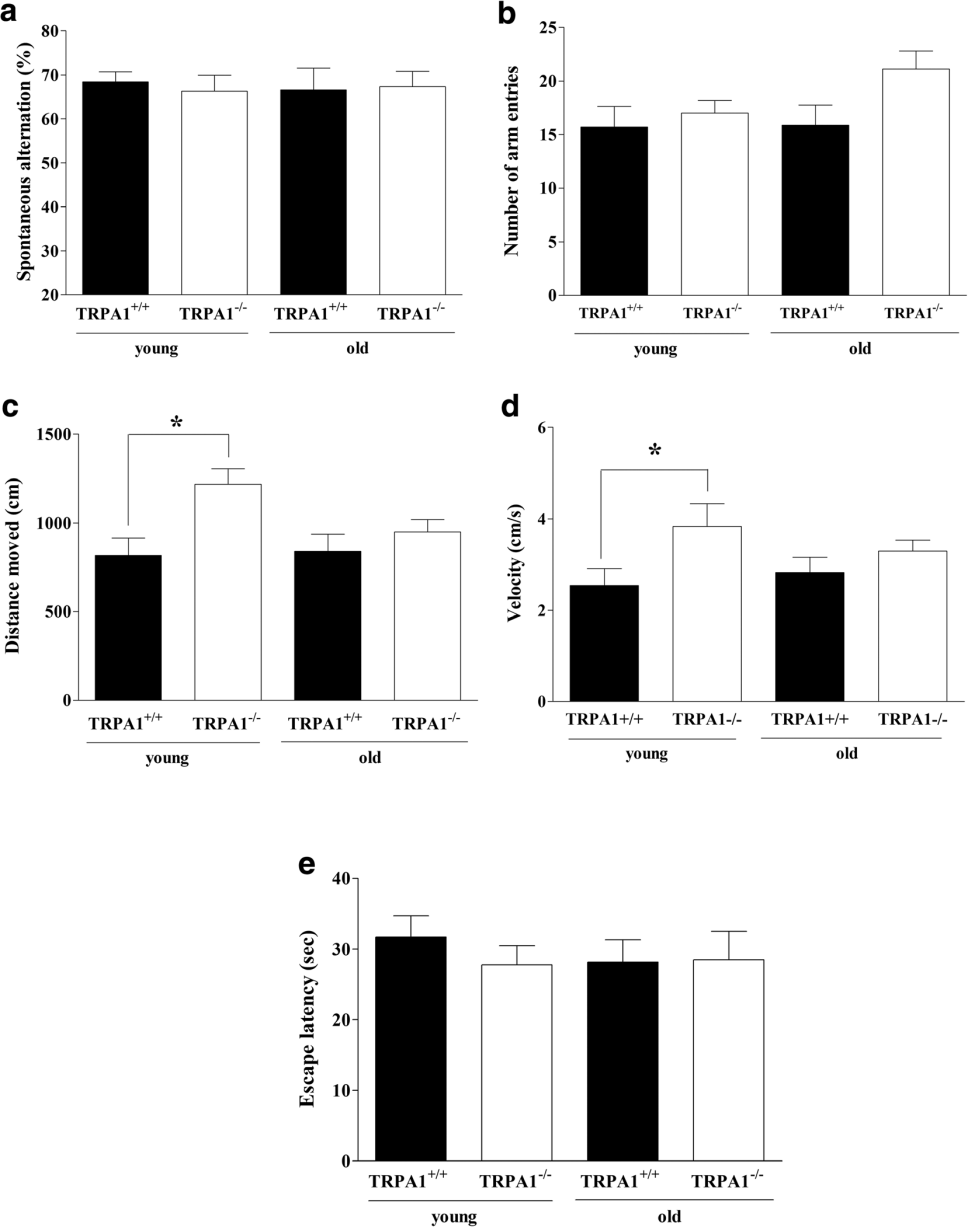
Age-dependent changes of the performance in the Y maze (YM) and Morris water maze (MWM) tasks in TRPA1^+/+^ and TRPA1^−/−^ young and old mice. Memory function was determined as spontaneous alternation (**a**), locomotor activity as number of arm entries (**b**), distance moved (**c**), and velocity (**d**) in YM. In the MWM, memory function was assessed as escape latency (**e**). Data are presented as the mean ± SEM (*n* = 6–10) and were analyzed by two-way ANOVA followed by Fischer’s posttest (*p < 0.05)

Spontaneous alternation measured in the Y maze was very similar in all investigated groups, approximately 67% (Fig. 4a). Furthermore, despite the higher mobility of the young TRPA1^−/−^ group (1218.00 ± 86.60 cm, 3.832 ± 0.5022 cm/s; Fig. 4c, d), which tendency was similar to the NOR test, the number of arm entries was very similar in all groups, approximately 16 (Fig. 4b).

Escape latency measured in the Morris water maze test did not show any significant difference neither between the old (TRPA1^+/+^ 28.15 ± 3.19; TRPA1^−/−^ 28.47 ± 4.04) and young groups (TRPA1^+/+^ 31.69 ± 3.04; TRPA1^−/−^ 27.78 ± 2.70) nor between WT and gene-deleted animals (Fig. 4e).

## Discussion

The present study has provided the first evidence that TRPA1 receptors are involved in senile memory loss of mice. We were able to assess memory decline with the NOR and RAM tests, while the spontaneous alternation in Y maze (YM) and the escape latency in Morris water maze (MWM) tests did not demonstrate the age-related downtrend of learning ability in our model.

TRPA1 is widely expressed throughout the body. It is localized in the peripheral, as well as the central nervous systems. There is strong evidence that TRPA1 plays a crucial role in pain transmission (Koivisto et al. 2018) and neuroimmune interactions (López-Requena et al. 2017). Therefore, it could be a potential drug target for several pain-related, respiratory, and vascular disorders (Nishida et al. 2015). However, studies of the last decade focused more and more to the CNS expressions and functions of TRPA1. There is an increasing evidence that TRPA1 can be found in the brain; both neuronal (cortex, hypothalamus, and hippocampus) and non-neuronal (astrocytes, Schwann cells, and endothelial cells) cell types express the receptor (Meents et al. 2019). Although our knowledge about TRPA1 functions in the CNS is limited (Sághy et al. 2016; Lee et al. 2017; Shigetomi et al. 2013; Lee et al. 2016), due to its polymodal nature, TRPA1 is very likely to be involved in numerous brain pathologies. A wide range of ligands, irritants, or stimulants can activate or sensitize the receptor (Nilius et al. 2012; Pozsgai et al. 2019; Chen and Hackos 2015). Furthermore, the consequence of TRPA1 activation can also be linked to CNS processes, because these receptors are highly permeable to calcium ions influencing calcium homeostasis of neurons and the glial cells (Hamilton et al. 2016).

Up to now, only few data have been published concerning the learning skills of TRPA1 gene-deleted animals. Memory functions of old (18 months) mice lacking TRPA1 have never been investigated, and only one recent paper describes behavioral and learning characteristics of young (8-week-old) TRPA1 knockout animals (Lee et al. 2017). In contrast to our results, they did not find any significant difference of motility in open field test. However, despite the variant protocol used in the NOR test, they also found that the discrimination index was higher in young mice lacking TRPA1 and the animals spent more time in the novel object zone. In our study, the same tendency could also be detected in old animals: aged WT mice showed marked memory loss and this alteration was significantly attenuated, nearly diminished in TRPA1^−/−^ animals. Both discrimination and recognition indexes, as well as the frequency of visiting the objects, were higher in knockouts. In the present study, the RAM test also indicated remarkable difference between the young WT and TRPA1 KO groups concerning memory function. Reference memory error was significantly lower in young animals lacking TRPA1 compared with the WTs. Furthermore, old WT animals needed significantly more time to find the rewards in the maze than TRPA1 knockouts, and only old WT animals found significantly less rewards compared with the young counterparts. These results clearly confirm the results obtained from a genetic model of Alzheimer's disease. TRPA1 depletion leads to significantly reduced memory loss in both models (Lee et al. 2016).

There are several various processes which can lead to age-related diseases. Due to the heterogeneity of the pathomechanisms, several mediators (pituitary adenylate cyclase-activating polypeptide, brain-derived neurotrophic factor, corticotropin-releasing factor, etc.) have recently been identified to be involved in the development of different disorders of aged animals/humans (Reglodi et al. 2018; Ungvari et al. 2017; Vedovelli et al. 2017; Fang et al. 2017; Tenk et al. 2017; Ashpole et al. 2017). Altered inflammatory responses and upregulation of genes encoding inflammatory mediators (Blalock et al. 2003; Hauss-Wegrzyniak et al. 2000; Andreasson et al. 2001; Franceschi and Campisi 2014), as well as cumulating oxidative stress and the oxidative damage of proteins (Carney et al. 1991; Davies et al. 2017; Tan et al. 2018), are well-known, important contributors to dementia. Similarly, altered mitochondrial function and calcium homeostasis, which are essentially responsible for the normal function of the brain cells (Alzheimer’s Association Calcium Hypothesis Workgroup 2017; Müller et al. 2018; Sure et al. 2018), can result in cell death and memory decline.

Our results fairly show that the lack of TRPA1 leads to significant attenuated memory loss in aged mice, suggesting that TRPA1 plays a crucial debating role in old age memory decline. According to the microarray data of the Human Brain Transcriptome Database and the Allen Brain Atlas, the TRPA1 expression in the human and murine brains is quite low, but it can be clearly detected. Its expression shows relatively high values in the temporal, prefrontal, primary auditory, and primary visual cortices and in the hippocampal area. Expression levels in the human brain do not change with age (Kang et al. 2011; Barrett et al. 2013; Hawrylycz et al. 2012). Although the underlying mechanisms are still unknown and need further investigations, several mediators involved in memory loss are known activators/sensitizers of TRPA1. We suggest that neuroinflammation and oxidative stress developing with age, together with increased vulnerability for oxidative stress in higher age (Carney et al. 1991), could lead to over-activation of the TRPA1 receptors. The increased Ca^2+^ influx to the cytoplasm, which alone, or together with potentiating the effects of other cation channels, like NMDA receptors, results in Ca^2+^ dysregulation of the cells leading to cell degeneration or cell death.

Another important aim of the study was to characterize the model. Aging of the animals leads to a much less robust memory loss than measured in other chemically or genetically induced dementia models. Therefore, it is difficult to find the most appropriate memory loss–sensitive behavioral tests. The NOR test is one of the most well-known methods for assessing memory function. Although the low exploration time should be considered a limitation of the NOR test, we demonstrated a significant decline of memory function in 18-month-old WT animals. Adequacy of the NOR test was also demonstrated in other accepted, oxidative stress (d-galactose, doxorubicin)–evoked animal models (Kaviani et al. 2017; Keeney et al. 2018). Radial arm water maze is a more widely applied test in aging/dementia studies compared with the RAM test (Buhusi et al. 2017; Sudduth et al. 2013); however, there are some data describing that dietary intake of natural toxins (Karlsson et al. 2009) or aging (Marighetto et al. 2008) can decrease learning ability measured by RAM test. It was absolutely a suitable method for the presentation of senile memory loss in the present study. Both working and reference memory errors increased significantly in aged mice compared with their younger counterparts. The exploration time was also longer in older animals, and old WT mice found significantly less rewards than young animals. In the present study, Y and Morris water maze tests did not show any difference between the young and old groups. The literature is also divergent in this question; the results are contradictory (Morgan et al. 2018; Hattori et al. 2016).

Nevertheless, dementia treatment is absolutely unsatisfactory: in most cases, only the mild cognitive decline can be corrected. Although several recommendations (including physical activity and diet) and programs (reality orientation training or cognitive stimulation therapy) are offered for patients with dementia in order to increase quality of life, there are no effective drugs on the market against severe memory loss. Since the TRPA1 receptor seems to be a key regulator of memory functions in younger and older ages in animal studies, receptor antagonists could open new perspectives in the pharmacotherapy of the senile dementia.
